# Supplementary material for: A Comparative Study of Tumor-Specificity and Neurotoxicity between 3-Styrylchromones and Anti-Cancer Drugs
Source: Medicines (Basel). 2023 Jul 14;10(7):43. doi: 10.3390/medicines10070043 (PMC10386476; doi:10.3390/medicines10070043)
Supplement: Supplementary file 1 [file medicines-10-00043-s001.zip › medicines-2461575-supplementary.pdf]

Supplementary Materials

Figure S1.
Induction of neurite extension in PC-12 cells by NGF. PC-12 cells were detached by trypsinization and incubated for 2 days to achieve complete adherence. Adherent cells were then incubated for 0, 2, 5 and 7 days in serum-free medium containing 50 ng/ml NGF with addition of fresh NGF solution at day 3 and day 6, using overlay method previously described (24).

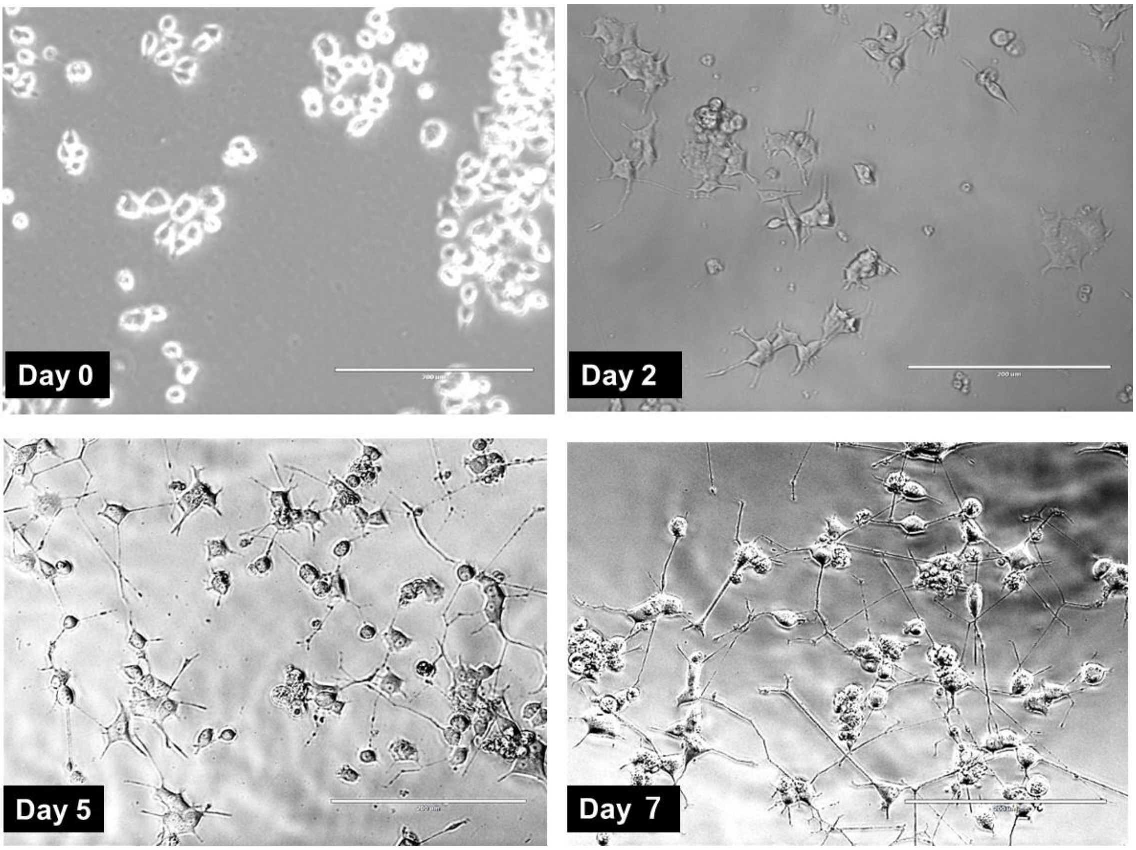

Table S1 Tumor-specificity of newly synthesized 65 chromone derivatives

	CC$_{50}$ (μM)										
	Human oral squamous cell carcinomaa cell lines					Human normal oral cells				TS	
	Ca9-22 (A)	HSC-2	HSC-3	HSC-4	mean (B)	HGF (C)	HPLF	HPC	mean (D)	(D/B)	(C/A)
**A 2- Indolylchromes (Series A)**											
**1**	**4.1**	**9.4**	**3.1**	**4.6**	**5.3**	**320.0**	**59.7**	**205.7**	**195.1**	**36.6**	**77.8**
2	64.9	35.5	30.3	13.8	36.2	43.5	16.8	29.5	29.9	0.8	0.7
3	75.1	62.5	157.0	70.6	91.3	269.7	174.7	262.0	235.4	2.6	3.6
4	26.3	23.0	171.3	183.0	100.9	202.0	60.0	148.3	136.8	1.4	7.7
5	8.6	5.9	8.2	16.9	9.9	88.6	38.9	87.0	71.5	7.2	10.4
6	17.7	12.4	10.9	18.2	14.8	170.3	167.0	163.7	167.0	11.3	9.6
7	6.3	30.1	29.8	52.8	29.7	272.3	202.3	238.0	237.6	8.0	43.1
8	3.1	3.1	3.2	3.8	3.3	35.3	10.5	10.9	18.9	5.7	11.4
**A**	**7.6**	**5.7**	**5.2**	**3.1**	**5.4**	**236.0**	**68.8**	**75.4**	**126.7**	**23.5**	**31.2**
DXR	0.35	0.09	0.11	0.08	0.16	>10	>10	4.6	>8.2	>52.3	>28.8
Melphalan	27.1	9.0	8.6	5.9	12.6	>200	186.0	165.0	>184	>14.5	>7.4
**B Indole-Aurone hybrid (Series B)**											
1	32.8	58.4	139.0	47.9	69.5	191.0	172.7	172.4	178.7	2.6	5.8
2	135.7	>400	337.9	>400	>318	379.5	231.6	237.1	282.7	<0.9	2.8
3	>400	177.8	252.2	>400	>307	260.7	123.5	179.1	187.8	<0.6	<0.7
4	48.0	>400	319.7	>400	>292	>400	250.0	390.1	>347	><1.2	>8.3
5	128.3	>400	295.5	49.8	>218	388.4	>400	>400	>396	><1.8	3.0
6	79.7	83.6	>400	172.9	>184	260.5	81.7	389.6	243.9	><1.3	3.3
7	95.0	>400	72.5	43.2	>153	279.7	25.7	250.3	185.3	<1.2	2.9
8	>400	>400	>400	>400	>400	>400	>400	261.6	>354	><0.9	><1
9	83.4	>400	>400	>400	>321	364.9	308.0	>400	>358	><1.1	4.4
**10**	**21.7**	**10.5**	**<3.13**	**10.1**	**11.3**	**>400**	**>400**	**>400**	**>400**	**>35.3**	**>18.5**
DOX	1.40	0.40	0.49	0.27	0.64	>10	8.1	>10	>9.4	>14.6	>7.2
**C Capsaicin derivatives (Series C)**											
1	>400	>400			>400	>400	>400		>400	><1	><1
2	>400	>400			>400	>400	>400		>400	><1	><1
3	154.2	178.9			166.6	387.9	292.0		339.9	2.0	2.5
4	121.4	94.4			107.9	>400	>400		>400	>3.7	>3.3
5	95.0	73.2			84.1	>400	>400		>400	>4.8	>4.2
**6**	**17.9**	**17.5**			**17.7**	**>400**	**>400**		**>400**	**>22.6**	**>22.3**
**7**	**14.8**	**10.6**			**12.7**	**>400**	**>400**		**>400**	**>31.5**	**>27.0**
8	61.7	62.3			62.0	98.3	93.2		95.8	1.5	1.6
9	31.8	17.4			24.6	167.7	58.3		113.0	4.6	5.3
10	43.3	26.1			34.7	83.8	78.8		81.3	2.3	1.9
11	140.9	131.0			136.0	282.9	258.8		270.9	2.0	2.0
12	>400	>400			>400	173.3	>400		>286.7	><0.7	<0.4
13	>400	>400			>400	250.0	>400		>325.0	><0.8	<0.6
14	164.3	195.0			179.65	35.4	200.0		117.7	0.7	0.2
15	71.7	70.8			71.25	5.8	53.6		29.7	0.4	0.1
16	200.0	>400			>300	169.2	>400		>285	><0.9	0.8
17	>400	>400			>400	4.3	>400		>202	><0.5	<0.01
18	>400	>400			>400	10.1	>400		>205	><0.5	<0.03
19	32.3	46.0			39.15	4.1	26.1		15.1	0.4	0.1
20	23.1	10.9			17	18.2	73.1		45.7	2.7	0.8
21	98.3	92.3			95.3	266.7	305.9		286.3	3.0	2.7
22	144.7	189.7			167.2	43.1	231.6		137.4	0.8	0.3
23	27.9	7.7			17.775	165.9	135.9		150.9	8.5	5.9
DXR	0.32	0.08			0.2015	>10	>10		>10	>49.6	>31.2
5-FU	72.9	150.0			111.45	>1000	>1000		>1000	>9.0	>13.7
Cisplatin	166.7	112.8			139.75	407.4	385.4		396.4	2.8	2.4
Melphalan	35.6	12.1			23.84	171.7	197.6		184.6	7.7	4.8

Table S1 (continued)

	CC50 (μM)										
	Human oral squamous cell carcinomaa cell lines					Human normal oral cells				TS	
	Ca9-22 (A)	HSC-2	HSC-3	HSC-4	mean (B)	HGF (C)	HPLF	HPC	mean (D)	(D/B)	(C/A)
**D 6,7-Styrylchromones (Series D)**											
1	25	19	117	22.7	45.9	164	>400		>282	>6.1	6.6
2	3.1	3.1	3.1	3.1	3.1	3.1	10.4		6.8	2.2	1.0
3	3.1	3.1	4.5	3.1	3.5	3.1	60		31.6	9.1	1.0
4	11.2	15.6	22.1	16.8	16.4	19.6	83		51.3	3.1	1.8
**5**	**3.9**	**3.1**	**14**	**4.2**	**6.3**	**3.1**	**>400**		**>201.6**	**>32.0**	**0.8**
6	5.6	5.3	16	7.4	8.6	5.8	79		42.4	4.9	1.0
7	13	14.3	22	18	16.8	19.1	30		24.6	1.5	1.5
8	8.3	9.8	13	11	10.5	20.8	27		23.9	2.3	2.5
9	3.6	3.1	4	5.4	4.0	4.6	100		52.3	13.0	1.3
10	5.5	3.1	18.1	5.7	8.1	9.4	100		54.7	6.8	1.7
11	3.4	3.1	7.2	5.2	4.7	3.1	67		35.1	7.4	0.9
12	3.5	3.1	5.5	5.5	4.4	3.1	92		47.6	10.8	0.9
DXR	0.45	0.17	0.31	0.17	0.28	1.3	6		3.7	13.3	2.9
5-FU	31	63	31	7.8	33.2	>1000	94		>547	>16.5	>32.3
Melphalan	41	14.8	19	8.5	20.8	168	186		177.0	8.5	4.1
**E 3-Benzylidenechromanones (Series E)**											
1	8.2	7.3	10.1	6.3	8.0	49.5	20.4	17.0	29.0	3.6	6.0
2	11.1	11.5	13.3	10.7	11.7	>400	20.3	38.4	>153	>13.1	>35.9
**3**	**2.8**	**4.1**	**2.2**	**5.3**	**3.6**	**>400**	**96.2**	**>400**	**>299**	**>82.6**	**>141.6**
4	6.6	7.7	6.3	8.9	7.4	49.5	11.5	21.2	27.4	3.7	7.5
5	4.0	4.5	9.8	8.2	6.6	100.0	363.6	28.1	163.9	24.8	25.2
6	31.3	40.3	44.6	43.1	39.8	87.5	183.3	109.1	126.6	3.2	2.8
7	16.6	11.4	18.9	9.2	14.0	>400	86.2	213.3	>233	>16.6	>24.2
8	6.0	5.3	7.5	6.3	6.3	>400	30.1	22.9	>151	>24.1	>66.3
9	4.3	5.1	7.2	4.4	5.3	>400	>400	37.5	>279	>53.1	>93.5
10	5.1	4.9	7.2	4.7	5.5	20.5	17.7	13.4	17.2	3.1	4.0
11	13.5	12.5	24.1	11.9	15.5	42.4	144.4	21.9	69.6	4.5	3.1
DOX	0.3	0.1	0.2	0.1	0.2	2.0	>10	>10	>7.3	>48.9	>6.7
5-FU	78.1	31.3	7.5	6.6	30.9	>1000	>1000	>1000	>1000	>37.9	>19.4
Cisplatin	79.3	62.5	14.0	24.0	45.0	727.3	219.3	108.9	351.8	7.8	9.2
Melphalan	40.8	14.0	16.7	11.1	20.6	192.5	>200	>200	>197	>9.6	>4.7

Table S2: Search for signaling pathway involved in 3-styrylchromone Induced-induced selective tumor-specificity against human OSCC cell lines

Compound #	1	2	3	4	5	6	7	8	9	10	11	12	13	14	
T-N	0.373	0.062	-0.159	0.523	-0.164	-0.001	2.373	0.566	0.501	0.522	0.190	0.862	0.503	2.086	*p*-value
**ERRPGC_ant (estrogen related receptor with PGC antagonist)**	**N**	**N**	**N**	**P**	**N**	**N**	**P**	**P**	**P**	**P**	**P**	**P**	**P**	**P**	**0.0307**
CAR_ant (constitutive androstane receptor antagonist)	N	N	N	N	N	N	N	P	N	N	N	P	N	P	0.1385
RAR_ant (retinoic acid receptor antagonist)	P	N	P	N	P	N	N	N	N	N	N	N	N	N	0.1472
ARant_ago (androgen receptor with antagonist agonist)	N	N	N	N	N	N	N	N	N	P	N	N	N	P	0.1567
TSHR_ago (thyroid stimulating hormone receptor agonist)	N	N	N	N	N	N	P	N	N	N	P	P	N	N	0.1619
H2AX_ago (histone variant H2AX agonist)	N	P	N	P	P	N	P	P	N	N	N	N	N	P	0.1825
GR_ant (glucocorticoid receptor antagonist)	P	P	P	N	N	N	N	N	P	N	N	N	N	N	0.2328
TRHR_ago (thyrotropin releasing hormone receptor agonist)	P	P	P	N	P	N	N	P	P	P	P	N	P	P	0.2913
TR_ant (thyroid receptor antagonist)	P	N	P	P	P	N	P	P	P	N	P	P	P	P	0.3295
PPARg_ant (peroxisome proliferator-activated receptor gamma antagonist)	N	N	N	N	N	N	N	N	N	N	P	P	P	P	0.3348
CaspC_ind (caspase-3/7 in CHO-K1 inducer)	P	N	P	N	P	N	P	P	P	N	P	P	P	P	0.3508
HDAC_ant (histone deacetylase antagonist)	P	P	P	P	P	N	N	P	N	N	P	N	P	P	0.3539
TRHR_ant (thyrotropin releasing hormone receptor antagonist)	N	N	N	N	N	P	N	N	N	N	N	N	N	P	0.3812
ERb_ant (estrogen receptor beta antagonist)	N	N	P	P	P	P	P	P	P	P	P	P	P	P	0.4774
RXR_ago (retinoid X receptor-alpha agonist)	N	P	N	N	N	N	N	N	N	N	N	N	N	N	0.4933
FXR_ago (farnesoid-X-receptor agonist)	P	P	P	P	N	P	N	P	N	N	P	P	P	P	0.5155
TSHR_ant (thyroid stimulating hormone receptor antagonist)	N	P	P	P	P	P	P	N	P	P	N	P	P	N	0.5234
ERRPGC_ago (estrogen related receptor with PGC agonist)	P	P	P	P	P	N	P	P	P	N	N	N	N	P	0.5461
TGFb_ant (transforming growth factor beta antagonist)	P	P	P	N	P	N	P	P	P	N	P	P	P	P	0.5573
CaspH_ind (caspase-3/7 in HepG2 inducer)	P	N	P	N	N	N	N	N	N	N	N	P	P	N	0.5673
ERlbd_ago (estrogen receptor alpha lbd agonist)	N	P	N	N	N	N	N	N	P	P	P	P	N	P	0.6411
ROR_ant (retinoid-related orphan receptor gamma antagonist)	P	P	P	P	P	P	P	P	P	N	N	P	P	P	0.6582
PPARd_ant (peroxisome proliferator-activated receptor delta antagonist)	N	N	N	N	P	N	P	P	P	P	P	P	P	N	0.6642
PR_ant (progesterone receptor antagonist)	P	P	P	P	P	P	P	P	P	P	P	N	P	P	0.724
ERsr_ago (endoplasmic reticulum stress response agonist)	P	N	P	P	P	P	P	P	P	P	P	N	P	P	0.8102
ARlbd_ant (androgen receptor lbd antagonist)	P	P	P	P	P	N	P	N	N	N	N	P	P	N	0.8227
ERR_ago (estrogen related receptor agonist)	P	P	P	P	P	P	P	P	P	N	N	N	N	P	0.8391
MMP_disr (mitochondrial membrane potential disruptor)	P	P	P	P	P	P	P	P	P	P	P	N	N	P	0.8582
ARfull_ant (androgen receptor full antagonist)	P	P	P	P	P	P	P	P	P	P	P	N	N	P	0.8582
GR_ago (glucocorticoid receptor agonist)	N	N	N	P	N	N	N	N	P	N	N	N	N	N	0.8849
HSR_act (heat shock response activator)	N	P	N	N	P	P	P	P	P	N	N	N	N	N	0.8966
CAR_ago (constitutive androstane receptor agonist)	P	P	P	P	P	P	P	P	P	P	P	P	N	P	0.9127
ARfulls_ant (androgen receptor with stimulator antagonist)	P	P	P	P	P	P	P	P	P	P	P	P	N	P	0.9127
ATAD5_ind (ATAD5 genotoxic inducer)	P	P	P	P	P	P	P	P	P	N	P	P	P	P	0.9318
NFkB_ago (NFkB agonist)	N	N	N	N	N	N	N	N	N	P	N	N	N	N	0.9318
AhR_ago (aryl hydrocarbon receptor agonist)	P	P	P	N	P	P	P	P	P	P	P	P	P	P	0.933
AP1_ago (activator protein-1 agonist)	P	P	P	P	N	N	P	P	P	N	N	N	N	N	0.9366
PPARg_ago (peroxisome proliferator-activated receptor gamma agonist)	P	P	P	N	N	N	N	P	N	N	N	N	P	P	0.9472
p53_ago (p53 agonist)	P	P	P	P	P	P	P	P	P	P	P	P	P	P	-
ARlbd_ago (androgen receptor lbd agonist)	N	N	N	N	N	N	N	N	N	N	N	N	N	N	-
ERlbd_ant (estrogen receptor alpha lbd antagonist)	P	P	P	P	P	P	P	P	P	P	P	P	P	P	-
ERfull_ant (estrogen receptor alpha full antagonist)	P	P	P	P	P	P	P	P	P	P	P	P	P	P	-
Arom_ant (aromatase antagonist)	P	P	P	P	P	P	P	P	P	P	P	P	P	P	-
ARE_ago (antioxidant response element agonist)	P	P	P	P	P	P	P	P	P	P	P	P	P	P	-
PPARd_ago (peroxisome proliferator-activated receptor delta agonist)	N	N	N	N	N	N	N	N	N	N	N	N	N	N	-
FXR_ant (farnesoid-X-receptor antagonist)	P	P	P	P	P	P	P	P	P	P	P	P	P	P	-
VDR_ago (vitamin D receptor agonist)	N	N	N	N	N	N	N	N	N	N	N	N	N	N	-
VDR_ant (vitamin D receptor antagonist)	P	P	P	P	P	P	P	P	P	P	P	P	P	P	-
HIF1_ago (hypoxia agonist)	N	N	N	N	N	N	N	N	N	N	N	N	N	N	-
ERfulls_ant (estrogen receptor alpha with stimulator antagonist)	P	P	P	P	P	P	P	P	P	P	P	P	P	P	-
Shh_ago (sonic hedgehog signaling agonist)	P	P	P	P	P	P	P	P	P	P	P	P	P	P	-
ERaant_ago (estrogen receptor alpha with antagonist agonist)	N	N	N	N	N	N	N	N	N	N	N	N	N	N	-
Shh_ant (sonic hedgehog signaling antagonist)	P	P	P	P	P	P	P	P	P	P	P	P	P	P	-
TSHR_agoant (thyroid stimulating hormone receptor agonist ・antagonist)	N	N	N	N	N	N	N	N	N	N	N	N	N	N	-
ERb_ago (estrogen receptor beta agonist)	N	N	N	N	N	N	N	N	N	N	N	N	N	N	-
ERR_ant (estrogen related receptor antagonist)	P	P	P	P	P	P	P	P	P	P	P	P	P	P	-
PXR_ago (human pregnane X receptor agonist)	P	P	P	P	P	P	P	P	P	P	P	P	P	P	-
TGFb_ago (transforming growth factor beta agonist)	N	N	N	N	N	N	N	N	N	N	N	N	N	N	-
PR_ago (progesterone receptor agonist)	N	N	N	N	N	N	N	N	N	N	N	N	N	N	-
